# Cental Macular Thickness in Patients with Type 2 Diabetes Mellitus without Clinical Retinopathy

**DOI:** 10.1155/2013/767931

**Published:** 2013-04-03

**Authors:** Mehmet Demir, Burcu Dirim, Zeynep Acar, Murat Yılmaz, Yekta Sendul

**Affiliations:** ^1^Department of Ophthalmology, Sisli Etfal Training and Research Hospital, Istanbul, Turkey; ^2^Sisli Etfal Hastanesi, Halaskargazi Caddesi Etfal Sokak No. 10, 34400 Sisli, Istanbul, Turkey; ^3^Department of Endocrinology, Namık Kemal University, Tekirdag, Turkey

## Abstract

*Objective*. To compare central macular thickness (CMT) of diabetic patients with type 2 diabetes without clinical retinopathy and healthy subjects. *Materials and Methods*. Optical coherence tomography (OCT) measurements were performed in 124 eyes of 62 subjects with diabetes mellitus without clinical retinopathy (study group: 39 females, 23 males; mean age: 55.06 ± 9.77 years) and in 120 eyes of 60 healthy subjects (control group: 35 females, 25 males; mean age: 55.78 ± 10.34 years). Blood biochemistry parameters were analyzed in all cases. The data for central macular thickness (at 1 mm), the levels of fasting plasma glucose, and glycosylated hemoglobin (HbA1c) were compared in both groups. *Results*. The mean central macular thickness was 232.12 ± 24.41 *µ*m in the study group and 227.19 ± 29.94 *µ*m in the control group. The mean HbA1c level was 8.92 ± 2.58% in the study group and 5.07 ± 0.70% in the control group (*P* = 0.001). No statistically significant relationship was found between CMT, HbA1c, and fasting plasma glucose level in either group (*P* > 0.05). *Conclusions*. Central macular thickness was not significantly thicker in patients with type 2 diabetes without clinical retinopathy than in healthy subjects.

## 1. Introduction

Diabetic retinopathy is the leading cause of blindness in adults in the working-age group in western countries. Diabetic macular edema (DME) has been reported at rates of 10% and occurs more frequently in type 2 diabetes mellitus than in type 1. Diabetic patients also have multiple risk factors for retinopathy, such as hyperglycemia and hypertension [1]. Their visual acuity is often dependent on the central foveal involvement, perifoveal capillary blood flow velocity, severity of perifoveal capillary occlusion, and retinal thickness at the central fovea [2, 3]. The clinical findings of diabetic retinopathy are microaneurysms, soft exudates, accumulation of hard exudates, and neovascularisation.

Macular edema can develop at any stage of diabetic retinopathy. In the past, macular edema was diagnosed with slit-lamp view. Fundus fluorescein angiography provides guidance for treatment of macular edema. Optical coherence tomography (OCT) has been used for detection of macular edema secondary to different pathologies, such as diabetes mellitus, central or branch retinal vein occlusion, uveitis, and age-related macular degeneration [4–11].

## 2. Materials and Methods

The central macular thickness (CMT) was measured in both groups by OCT (Optovue Inc. Co., RTVue 100 model, Fremont, CA, USA). The CMT was measured after providing pupil dilation with tropicamide drops 2 times, 10 minutes before measurements (Tropicamide 1%, Alcon Lab. Inc., USA). Three measurements were taken from each patient after pupillary dilatation. Blood biochemical tests for glycosylated hemoglobin (HbA1c) and fasting plasma glucose levels were run on all patients. All cases underwent ophthalmological examinations including best corrected visual acuity (BCVA), anterior and posterior segment examinations under slit lamp, intraocular pressure (IOP) (applanation tonometer model AT 900, Haag-Streit, Switzerland), and central macular thickness measured by OCT. Visual acuity was measured with an early treatment diabetic retinopathy study chart at 4 meters. Each subject gave a written informed consent to participate in the study. The study adhered to the tenets of the Declaration of Helsinki.

The study group included 62 patients (124 eyes; 39 females, 23 males; mean age: 55.06 ± 9.77 years) who had type 2 diabetes mellitus without clinical retinopathy, and the control group included 60 patients (120 eyes; 35 females, 25 males; mean age: 55.78 ± 10.34 years) (Table 1). Inclusion criteria for the study group included no visible findings of diabetic retinopathy (hard-soft exudates, microaneurysms) on retina at slit-lamp fundus examination with a +78 D lens, type 2 diabetes mellitus, no other problems (such as hypertension, uveitis), and no history of ophthalmologic trauma, intravitreal injection, high refractive errors (spherical equivalent between +1.00 D and −1.00 D), or use of drugs(s) for retinal problems. Inclusion criteria for the control group patients included no ophthalmologic or systemic problems, no history of intraocular surgery or treatment of the retina, and no high refractive errors (spherical equivalent: between −1.0 D and +1.0 D). Exclusion criteria for both groups were visible retinopathy or uveitis, hypertension, or previous ophthalmologic surgery. In the study group, the duration of diabetes mellitus ranged from 0 to 20 years, and the average was 7.19 ± 4.87 years. Five patients were newly diagnosed, 19 patients had been diagnosed for 1–5 years, 23 patients had been diagnosed for 6–10 years, 9 patients had been diagnosed for 11–15 years, and 6 patients had been diagnosed for more than 15 years. In the study group, five patients were newly diagnosed, 49 patients were undergoing insulin treatment, and 8 patients were taking oral antidiabetic drugs (Table 2). Both groups were compared based on mean age, central macular thickness, fasting plasma glucose, and HbA1c levels.

## 3. Statistical Analysis

The Number Cruncher Statistical System (NCSS) 2007 and the PASS 2008 statistical software (Utah, USA) programs were used to evaluate the results of the study. 

Descriptive statistical methods (mean, standard deviation) and Student's *t*-test were used together to compare the data from the two groups and the parameters that showed normal distribution. The Mann-Whitney *U* test was used to compare parameters of the two groups that did not show normal distribution. A chi-square test was used to compare the quality of the data. Pearson's correlation analyses were conducted to evaluate the relationship between the parameters showing normal distribution, and Spearman's rho correlation analyses have been used to evaluate the correlation between the parameters not showing normal distribution. A value of *P* < 0.05 was considered significant.

## 4. Results

Best corrected visual acuity (BCVA) was 0.00 logMAR in both groups. No significant differences were found for the mean age, IOP, or gender distribution (Table 1).

The mean HbA1c level was 8.92 ± 2.58% in the study group and 5.07 ± 0.70% in the control group. The mean level of HbA1c was statistically higher in the study group than in the control group (Table 1, *P* = 0.001). Fasting plasma glucose level was statistically higher in the study group than in the control group (Table 1, *P* = 0.001). The duration of diabetes mellitus was 7.19 ± 4.8 (range 0–20) years. The mean of CMT was 232.12 ± 24.41 *μ*m in the study group and 227.19 ± 29.94 *μ*m in the control group (Table 1). The CMT was thicker in the study group than in the control group, but this difference was not statistically significant.

No relationship was found between CMT and fasting plasma glucose level in the study (*P* = 0.483) and control (*P* = 0.399) groups. No relationship was found between CMT and HbA1c level in the study (*P* = 0.550) and control groups (*P* = 0.997; Table 3).

## 5. Discussion

We found no studies in the literature which reviewed CMT, fasting plasma glucose level, and level of HbA1c less than HbA1c 8%.

Several previous studies by Udaondo et al. [12], Moreira et al. [13], Schneeberg and Göbel [14], Song et al. [15], Takatsuna et al. [16], and Vemala et al. [17] determined that optical coherence tomography can help in the evaluation of macular edema in diabetic or nondiabetic patients and also help in the followup of the patients during treatment to establish quantitative or qualitative responses to therapy.

We reviewed the relationship between central macular thickness, HbA1c, and fasting plasma glucose levels in patients with type 2 diabetes without clinical diabetic retinopathy. Optical coherence tomography (OCT) was used for objective measurement and monitoring of central macular thickness. Browning et al. [18] and Hee et al. [19], described that a change in the OCT measurements greater than 10% of the baseline thickness is likely to represent a true change in macular thickness. Glycosylated hemoglobin is a parameter that can be used to follow up hyperglycemia over the long term. Moon et al. [20] suggested that a high baseline HbA1c and a large reduction in HbA1c were risk factors for the increase in macular thickness. Yeung et al. [21] showed that HbA1c level positively correlated with macular thickness in patients with type 1 and 2 diabetes of 10 or more years' duration without diabetic macular edema. Chou et al. [22] showed that a HbA1c level of 8% or above was associated with an increase in macular thickness in diabetic patients with diabetic retinopathy. Yeung et al. [21], Chou et al. [22], and Rosenstock et al. [23] concluded that meticulous diabetes control may slow the progression of early diabetic retinopathy and may play an important role in preventing macular dysfunction. In type 1 and 2 diabetes patients, strict followup of plasma glucose level could reduce the progression and development of diabetic retinopathy.

The purpose of this study was to examine central macular thickness in patients with type 2 diabetes mellitus without retinopathy. This study showed the following four results. (1) The mean central macular thickness is thicker in diabetic patients without diabetic retinopathy than in healthy subjects, but this difference was not statistically significant. (2) No positive relationship was found between fasting plasma glucose level and the central macular thickness in patients with diabetes mellitus without retinopathy. (3) Central macular thickness was not increased by mild or high levels of HbA1c (8.92 ± 2.59%). (4) Central macular thickness was not affected by the duration of diabetes mellitus in patients with diabetes type 2 without retinopathy. There are limitations to our study. One of these is the small sample size in both groups and another is that no patients had diabetes mellitus for longer than 20 years.

## 6. Conclusion

Our opinion is that the truly effective parameter on macular thickness is vascular permeability in patients with diabetes mellitus.

In this study, glycosylated HbA1c and fasting plasma glucose levels were significantly higher in diabetic patients without retinopathy than in the control group, although there was no difference in central macular thickness between the two groups.

## Figures and Tables

**Table 1:** Demographic characteristics, values for central macular thickness (CMT), and biochemical analysis in patients with type 2 diabetes without clinical retinopathy.

Parameters	Study group (*n* = 62)	Control group (*n* = 60)	*P*
BCVA	0.00 (logMAR)	0.00 (logMAR)	NS
IOP mmHg	17,8 ± 2.3 mmHg	18.1 ± 2.1 mmHg	NS
Age (mean ± SD)	55.06 ± 9.77	55.78 ± 10.34	NS
Male/female	23/39	25/35	NS
CMT *µ*m (±SD)	232.12 ± 24.41	227.19 ± 29.94	NS
HbA1c (mean ± SD)	8.92 ± 2.58	5.07 ± 0.70	0.001

Fasting blood glucose level			
Average	202.14 ± 104.78 (median: 178)	92.17 ± 7.75 (median: 92)	0.001

BCVA: best corrected visual acuity, IOP mmHg: mean Intraocular pressure, millimeter mercury, CMT: central macular thickness, µm: micrometer, SD: standard deviation, logMAR: logarithm of the minimum angle of resolution, HbA1c: glycosylated hemoglobin, *n*: number of patients, logMAR: logarithm of the minimum angle of resolution, NS: nonsignificant, S: significant (*P* < 0.05), study group: patients with type 2 diabetes without clinical retinopathy; control group: healthy subjects.

**Table 2:** Duration and treatment of diabetes mellitus in patients with type 2 diabetes without clinical retinopathy.

Duration of DM	(*n* = 62)	%
New diagnosis	5	8.1
1–5 years	19	30.6
6–10 years	23	37.1
11–15 years	9	14.5
>15 years	6	9.7
Insulin treatment	49	79
OAD	8	12.9

DM: diabetes mellitus, *n*: number of patients, and OAD: oral antidiabetic drugs.

**Table 3:** Relationship between central macular thickness (CMT), glycosylated hemoglobin (HbA1c), and fasting blood glucose levels in patients with type 2 diabetes without clinical retinopathy.

Parameters	Study group		Control group	
	*r*	*P*	*r*	*P*
CMT-HbA1c	−0.077	NS	0.001	NS
CMT-fasting glucose	−0.091	NS	0.011	NS

CMT: central macular thickness, HbA1c: glycosylated hemoglobin, *P*: statistic value, and *r*: relation between two variables. NS: nonsignificant, study group: patients with type 2 diabetes without clinical retinopathy, and control group: healthy subjects.

## References

[1] Girach A, Lund-Andersen H (2007). Diabetic macular oedema: a clinical overview. *International Journal of Clinical Practice*.

[2] Gardner TW, Larsen M, Girach A, Zhi X (2009). Diabetic macular oedema and visual loss: relationship to location, severity and duration. *Acta Ophthalmologica*.

[3] Sakata K, Funatsu H, Harino S, Noma H, Hori S (2007). Relationship of Macular Microcirculation and Retinal Thickness with Visual Acuity in Diabetic Macular Edema. *Ophthalmology*.

[4] Yahia SB, Kahloun R, Jelliti B, Khairallah M (2011). Branch retinal artery occlusion associated with Behçet disease. *Ocular Immunology and Inflammation*.

[5] Ogino K, Tsujikawa A, Nakamura H (2011). Focal macular electroretinogram in macular edema secondary to central retinal vein occlusion. *Investigative Ophthalmology & Visual Science*.

[6] Browning DJ, McOwen MD, Bowen RM, O’Marah TL (2004). Comparison of the clinical diagnosis of diabetic macular edema with diagnosis by optical coherence tomography. *Ophthalmology*.

[7] Virgili G, Menchini F, Murro V, Peluso E, Rosa F, Casazza G (2011). Optical coherence tomography (OCT) for detection of macular oedema in patients with diabetic retinopathy. *Cochrane Database of Systematic Reviews*.

[8] Ibrahim MA, Sepah YJ, Symons RC, Channa R, Hatef E, Khwaja A (2012). Spectral- and time-domain optical coherence tomography measurements of macular thickness in normal eyes and in eyes with diabetic macular edema. *Eye*.

[9] Medina FJ, Callén CI, Rebolleda G, Muٌoz-Negrete FJ, Callén MJ, Valle FG (2012). Use of nonmydriatic spectral-domain optical coherence tomography for diagnosing diabetic macular edema. *The American Journal of Ophthalmology*.

[10] Suzuma K, Yamada Y, Liu M, Tsuiki E, Fujikawa A, Kitaoka T (2011). Comparing central retinal thickness in diabetic macular edema measured by two different spectral-domain optical coherence tomography devices. *The Japanese Journal of Ophthalmology*.

[11] Kwon SI, Hwang DJ, Seo JY, Park IW (2011). Evaluation of changes of macular thickness in diabetic retinopathy after cataract surgery. *The Korean Journal of Ophthalmology*.

[12] Udaondo P, Díaz-Llopis M, García-Delpech S, Salom D, Romero FJ (2011). Intravitreal plasmin without vitrectomy for macular edema secondary to branch retinal vein occlusion. *Archives of Ophthalmology*.

[13] Moreira RO, Trujillo FR, Meirelles RMR, Ellinger VCM, Zagury L (2001). Use of optical coherence tomography (OCT) and indirect ophthalmoscopy in the diagnosis of macular edema in diabetic patients. *International Ophthalmology*.

[14] Schneeberg AE, Göbel W (2003). Diagnosis and Follow-up of non-diabetic Macular Edema with the Optical Coherence Tomography (OCT). *Ophthalmologe*.

[15] Song JH, Lee JJ, Lee SJ (2011). Comparison of the short-term effects of intravitreal triamcinolone acetonide and bevacizumab injection for diabetic macular edema. *The Korean Journal of Ophthalmology*.

[16] Takatsuna Y, Yamamoto S, Nakamura Y, Tatsumi T, Arai M, Mitamura Y (2011). Long-term therapeutic efficacy of the subthreshold micropulse diode laser photocoagulation for diabetic macular edema. *The Japanese Journal of Ophthalmology*.

[17] Vemala R, Koshy S, Sivaprasad S (2011). Qualitative and quantitative OCT response of diffuse diabetic macular oedema to macular laser photocoagulation. *Eye*.

[18] Browning DJ, Fraser CM, Propst BW (2008). The Variation in Optical Coherence Tomography-Measured Macular Thickness in Diabetic Eyes Without Clinical Macular Edema. *The American Journal of Ophthalmology*.

[19] Hee MR, Puliafito CA, Wong C (1995). Quantitative assessment of macular edema with optical coherence tomography. *Archives of Ophthalmology*.

[20] Moon SW, Kim HY, Kim SW, Oh J, Huh K, Oh IK (2011). The change of macular thickness measured by optical coherence tomography in relation to glycemic control in diabetic patients. *Graefe’s Archive for Clinical and Experimental Ophthalmology*.

[21] Yeung L, Sun CC, Ku WC (2010). Associations between chronic glycosylated haemoglobin (HbA1c) level and macular volume in diabetes patients without macular oedema. *Acta Ophthalmologica*.

[22] Chou TH, Wu PC, Kuo JZC, Lai CH, Kuo CN (2009). Relationship of diabetic macular oedema with glycosylated haemoglobin. *Eye*.

[23] Rosenstock J, Friberg T, Raskin P (1986). Effect of glycemic control on microvascular complications in patients with type I diabetes mellitus. *The American Journal of Medicine*.

